# Preliminary observations on soluble programmed cell death protein-1 as a prognostic and predictive biomarker in patients with metastatic melanoma treated with patient-specific autologous vaccines

**DOI:** 10.18632/oncotarget.27164

**Published:** 2019-09-03

**Authors:** Robert O. Dillman, Gabriel I. Nistor, Bryce T. McLelland, Candace Hsieh, Aleksandra J. Poole, Andrew N. Cornforth, Hans S. Keirstead

**Affiliations:** ^1^ AIVITA Biomedical, Inc. Irvine, CA 92612, USA; ^2^ Hoag Cancer Institute, Newport Beach, CA 92663, USA

**Keywords:** programmed cell death protein -1 (PD-1), dendritic cell vaccines, metastatic melanoma

## Abstract

Because of its role as an immune checkpoint, levels of soluble programmed cell death protein-1 (sPD-1) could be useful as a prognostic biomarker or predictive biomarker in cancer patients treated with vaccines. Very low levels of sPD-1 may indicate lack of an existing anti-cancer immune response; very high levels may indicate an active immune response that is suppressed. In between these extremes, a decrease in PD-1 following injections of an anti-cancer vaccine may indicate an enhanced immune response that has not been suppressed. Blood samples obtained during a randomized trial in patients with metastatic melanoma were tested from 22 patients treated with a tumor cell vaccine (TCV) and 17 treated with a dendritic cell vaccine (DCV). Survival was better in DCV-treated patients. sPD-1 was measured at week-0, one week before the first of three weekly subcutaneous injections, and at week-4, one week after the third injection. The combination of a very low baseline sPD-1, or absence of a very high PD-1 at baseline followed by a decline in sPD-1 at week-4, was predictive of surviving three or more years in DCV-treated patients, but not TCV-treated. Among DCV-treated patients, these sPD-1 criteria appropriately classified 8/10 (80%) of 3-year survivors, and 6/7 (86%) of patients who did not survive three years. These preliminary observations suggest that sPD-1 might be a useful biomarker for melanoma patients being considered for treatment with this DCV vaccine, and/or to predict efficacy after only three injections, but this would have to be confirmed in larger studies.

## INTRODUCTION

PD-1 (CD279) was first described by Tasuku Honjo and colleagues at Kyoto University in 1992 [1]. Dr. Honjo was awarded a Nobel Prize in 2018 for his pioneering studies of PD-1, including its expression on immune cells, and characterization of its role in regulating immune responses via interaction with specific ligands (PD-L1 and PD-L2) [2–7]. Because of its role as an immune checkpoint, levels of soluble programmed cell death protein-1 (sPD-1) could be useful as a prognostic biomarker or predictive biomarker in cancer patients treated with vaccines because it is upregulated on activated lymphocytes by interferon gamma, during a Th1 immune response [6, 8–10]. In cancer patients, very low levels of sPD-1 may indicate lack of an existing anti-cancer immune response while very high levels may indicate an active immune response that has been suppressed. In between these extremes, a decrease in PD-1 following injections of an anti-cancer vaccine may indicate an enhanced immune response that has not been suppressed.

The purpose of this study was to determine the possible use of sPD-1 as an immune marker in patients with metastatic melanoma who were enrolled in a randomized phase II trial testing autologous dendritic cell vaccines (DCV) and autologous tumor cell vaccines (TCV) [11, 12]. In particular, we were looking for a surrogate marker that might reflect immune response and association with survival in patients treated with these vaccines. For both products, the antigen source was irradiated autologous cancer cells from short-term cell cultures derived from surgically excised autologous tumor. We asked the following questions: (1) was baseline sPD-1 prognostic for survival in these patients with metastatic melanoma, or for either of the two treatment-defined cohorts; (2) within each treatment-defined cohort, was either vaccine efficacious in patients with very low sPD-1 levels and/or very high sPD-1 levels; (3) if there was a change in sPD-1 from week-0, one week before the first of three weekly vaccine injections, to week-4, one week after the third injection, was this predictive of survival for all patients or in either treatment-defined cohort; and (4) could sPD-1 be used to define cohorts that were prognostic or predictive of survival.

## RESULTS

### Summary data

**Figure 1:**
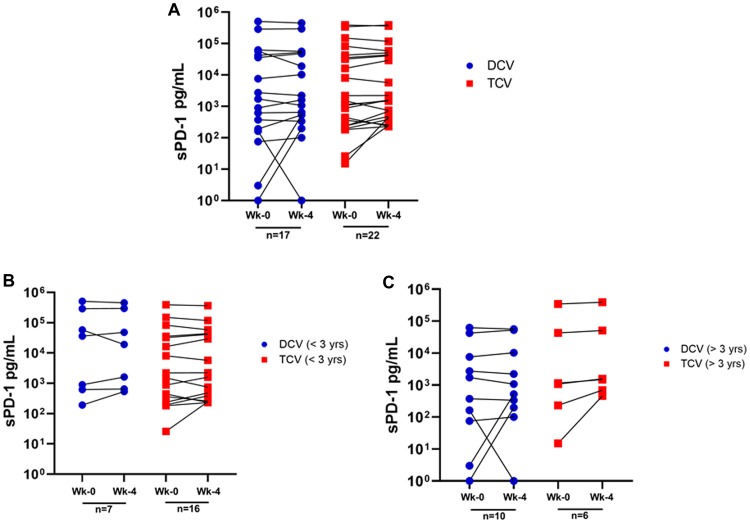
Changes in sPD-1 between week-0 and week-4 for individual patients. (**A**) shows all 39 patients by treatment arm - dendritic cell vaccine (DCV) or tumor cell vaccine (TCV); (**B**) shows the 23 patients who survived less than three years by treatment arm (DCV or TCV); and (**C**) shows the 16 patients who survived more than three years by treatment arm (DCV or TCV).

Tables 1 and 2 display the sPD-1 data for all 39 patients. Table 1 displays the week-0 baseline sPD-1, week-4 sPD-1, absolute and percentage changes in sPD-1 from week-0 baseline to week-4, and actual survival for the DCV-treated patients. Table 2 displays similar data for TCV-treated patients. In addition, Figure 1 shows the changes in sPD-1 levels graphically for each individual patient by treatment arm (Figure 1A), and by subcohorts based on 3-year survival within each group (Figures 1B and 1C). Table 3 shows the median and mean sPD-1 levels for week-0 and week-4 and changes in the mean and median sPD-1 levels for all 39 patients as a group, as well as cohorts defined by treatment (DCV or TCV) and/or survival (greater or less than three years).

**Table 1:** Autologous dendritic cell vaccine (DCV): soluble programmed death protein-1 (sPD-1) levels before and after three weekly injections of DCV and associated survival in patients with metastatic melanoma

Patient Number	Week-0 sPD-1 (pg/mL)	Week-4 sPD-1 (pg/mL)	Change in sPD-1 (pg/mL)	% Change	Survival (months)
1	1	198	198	19,700.0%	42.2
2	3	520	517	17,233.3%	44.6
3	75	100	25	34.1%	60+
4	164	0	−164	–100.0%	60+
5	191	532	341	178.5%	19.1
6	372	335	−37	−9.9%	52.9
7	618	650	32	5.2%	25.2
8	897	1,613	716	79.8%	13.0
9	1,733	1,078	−655	–37.8%	53.0
10	2,723	2,226	−497	–18.2%	60+
11	7,645	10,340	2,695	35.3%	60+
12	36,277	48,001	11,724	32.3%	17.6
13	42,393	52,570	10,177	24.0%	60+
14	57,687	19,160	–38,527	−66.8%	18.6
15	62,777	56,841	−5,936	−9.5%	38.6
16	290,060	298,072	8,012	2.8%	7.9
17	511,063	455,212	−55,851	–10.9%	9.6
18	–	–	–	–	60+

sPD-1= soluble PD-1; pg/mL=picogram/milliliter

**Table 2:** Autologous tumor cell vaccine (TCV): soluble programmed death protein-1 (sPD-1) levels before and after three weekly injections of TCV and associated survival in patients with metastatic melanoma

Patient Number	Week-0 sPD-1 (pg/mL)	Week-4 sPD-1 (pg/mL)	Change in sPD-1 (pg/mL)	% change	Survival (months)
1	15	462	447	96.8%	60+
2	26	247	221	89.5%	30.3
3	183	230	47	20.4%	4.0
4	193	388	195	50.3%	9.3
5	233	694	461	66.4%	60+
6	238	481	243	50.5%	13.2
7	364	259	-105	−40.5%	33.7
8	446	234	-212	−90.6%	32.2
9	877	1,591	714	44.9%	3.9
10	1,075	1,584	509	32.1%	60+
11	1,152	1,488	336	22.6%	60+
12	1,509	745	−764	-102.6%	9.0
13	2,201	2,238	37	1.7%	16.9
14	8,121	5,710	-2,411	95.3%	9.9
15	16,277	29,276	12,999	−42.2%	14.6
16	32,287	41,123	8,836	44.4%	21.7
17	35,637	43,826	8,189	21.5%	2.5
18	43,095	50,939	7,844	18.7%	60+
19	82,755	58,223	-24,532	15.4%	19.9
20	150,588	118,159	-32,429	-27.4%	32.3
21	343,919	389,327	45,408	11.7%	60+
22	392,201	364,014	-28,187	−7.7%	21.1
23	–	–	–	–	0.7
24	–	–	–	–	1.1

sPD-1 = soluble PD-1; pg/mL = picogram/milliliter.

**Table 3:** Soluble programmed death protein-1 (sPD-1) levels in metastatic melanoma patients enrolled in a randomized trial

Population	Number of Patients	Median sPD-1 (pg/mL)	Mean sPD-1 (pg/mL)	St Dev sPD-1 (pg/mL)	Change Mean Wk-0 to Wk-4 (pg/mL)	Change Median Wk-0 to wk-4 (pg/mL)
All Wk-0	*N* = 39	1,509	54,566	119,845	−1779	+82
All Wk-4	*N* = 39	1,591	52,787	115,284	(−3.3%)	(+5.4%)
**> 3 yr survival**						
Wk-0	*n* = 16	1,114	31,711	85,580	+3,833	+170
Wk-4	*n* = 16	1,283	35,544	96,663	(+12.1%)	(+15.2%)
**< 3 yr survival**						
Wk-0	*n* = 23	2,201	70,465	138,459	−5,683	+37
Wk-4	*n* = 23	2,238	64,782	127,312	(−8.1%)	(+1.7%)
**Treatment arm**						
TCV Wk-0	*n* = 22	1,331	50,609	109,063	−98	+257
TCV Wk-4	*n* = 22	1,588	50,511	109,634	(−0.2%)	(+19.3%)
DCV Wk-0	*n* = 17	1,733	59,687	135,833	–3,955	−120
DCV Wk-4	*n =* 17	1,613	55,732	125,510	(−6.6%)	(−6.9%)
**Treatment arm and survival**						
TCV OS	< 3 yrs					
Wk-0	*n =* 16	1,855	45,244	101,076	–3,572	+60
Wk-4	*n =* 16	1,915	41,672	91,864	(–7.9%)	(+3.2%)
TCV OS	> 3 yrs					
Wk-0	*n =* 6	1,114	64,915	137,736	+9,167	+423
Wk-4	*n =* 6	1,536	74,082	155,722	(+14.1%)	(+37.9%)
DCV OS	< 3 yrs					
Wk-0	*n =* 7	36,277	128,113	197,937	–10,508	–17,117
Wk-4	*n =* 7	19,160	117,606	183,449	(–8.2%)	(–47.2%)
DCV OS	> 3 yrs					
Wk-0	*n =* 10	1,053	11,788	22,155	+632	–254
Wk-4	*n =* 10	799	12,421	22,522	(+5.4%)	(–24.1%)

sPD-1= soluble programmed death protein-1. Data shown is for all 39 patients

(All) and for two treatment-defined cohorts: TCV = autologous tumor cell vaccine (TCV) (*n* = 22); DCV = autologous dendritic cell vaccine (DCV) (*n* = 17). OS = overall survival. Subcohorts were defined by treatment arm and survival greater than (>) or less than (<) three years (3 yrs). Grouped sPD-1 levels were measured at baseline, one week before starting vaccine therapy (wk-0), and four weeks later (wk-4), after the first three vaccine injections.

### Baseline sPD-1 levels were not prognostic for survival

**Figure 2:**
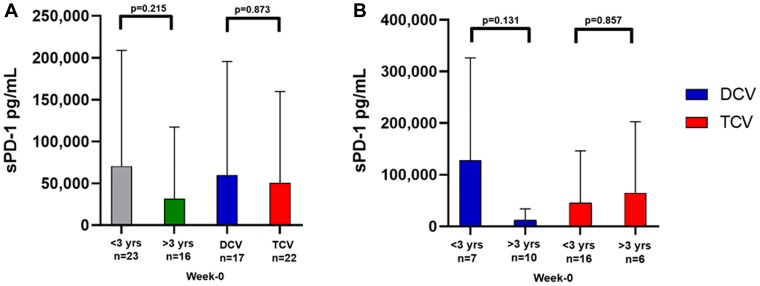
Mean levels of baseline sPD-1 in various cohorts. (**A**) shows there was no difference in sPD-1 levels (Mann–Whitney *U* Test) by survival less than three years versus greater than three years, or DCV versus TCV treatment arm. (**B**) shows a trend toward higher sPD-1 levels in DCV-treated patients who survived less than three years compared to those who survived more than three years (*p =* 0.131 Mann–Whitney *U*-Test) This was not observed in the TCV-treated patients (*p =* 0.857 Mann–Whitney *U*-Test). sPD-1= soluble programmed cell death protein-1, DCV=dendritic cell vaccine, TCV=tumor cell vaccine. Data are presented as mean ± SD.

The mean sPD-1 level for three healthy controls was 595 pg/mL, which is in the normal range of 200 to 1200 pg/mL reported by others [13–21]. For all 39 patients, baseline sPD-1 levels ranged from 0 to 511,063 pg/mL (Tables 1 and 2) with a mean of 54,566 pg/mL and median of 119,845 pg/mL (Table 3). There was no difference in the means of baseline sPD-1 for the 17 DCV-treated compared to the 22 TCV-treated (Table 3, Figure 2A). There was also no difference in the means of baseline sPD-1 for the 16 patients who survived greater than three years compared to the 23 who survived less than three years (Table 3, Figure 2A). The most striking variation in Table 3 is the high mean week-0 sPD-1 levels for the seven DCV-treated patients who survived less than three years. This difference is shown graphically in Figure 2B and contrasted to the low sPD-1 levels recorded at baseline in the 10 DCV-treated patients who survived more than three years, and in TCV-treated patients. However, because of the wide variation in values and the relatively small numbers of patients, this great difference in the mean baseline sPD-1 levels in the 10 DCV-treated patients who survived greater than three years was not significantly different compared to the sPD-1 levels for the 7 DCV-treated patients who survived less than three years (*p =* 0.131) (Table 3, Figure 2B). Among TCV-treated patients, the median and mean baseline sPD-1 levels were similar for the 16 who survived less than three years and for the six who survived greater than three years (Table 3, Figure 2B).

**Figure 3:**
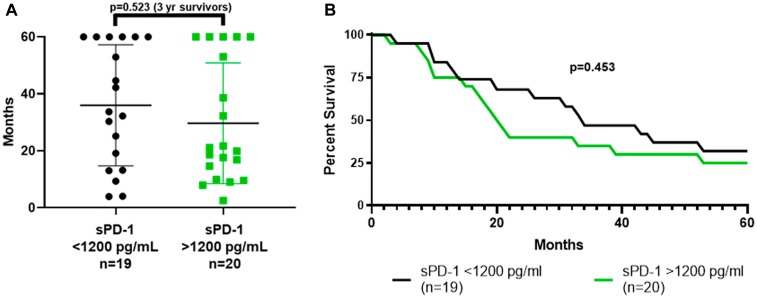
Survival based on whether baseline sPD-1 level was above or below 1,200 pg/mL. (**A**) dot plot shows the distribution of survival for the 19 patients who had a baseline sPD-1 level less than 1,200 pg/mL, and for the 20 patients who had a baseline sPD-1 level greater than 1,200 pg/mL. The proportions who survived more than three years, 9/19 vs 7/20, did not differ (*p =* 0.523, Fisher Exact Test). (**B**) shows actual survival curves (all patients followed to death or five years with none lost to follow-up). There was no difference between the curves (*p =* 0.453, Mantel-Haenszel log rank-test). sPD-1 = soluble programmed cell death protein-1.

Patients were grouped by sPD-1 levels that were less than or greater than 1,200 pg/mL, which provided the same distribution on either side of the median of 1,509 for all 39 patients (Tables 1–3). The proportion surviving greater than three years was no greater for the 19 patients with baseline sPD-1 less than 1,200 pg/mL compared to the 20 patients with baseline sPD-1 greater than 1,200 pg/mL (9/19 versus 7/20, *p =* 0.523) (Figure 3A). In addition, overall survival (OS) was no better for 19 patients with baseline sPD-1 less than 1,200 pg/mL compared to 20 patients with sPD-1 greater than 1,200 pg/mL (median OS 33.0 vs 19.9 mos.; 3-yr OS 47% vs 35%, *p =* 0.453) (Figure 3B). Thus, baseline sPD-1 was not a prognostic marker for survival for this population of melanoma patients.

### Changes in sPD-1 levels from week-0 to week-4 were not predictive of survival

**Figure 4:**
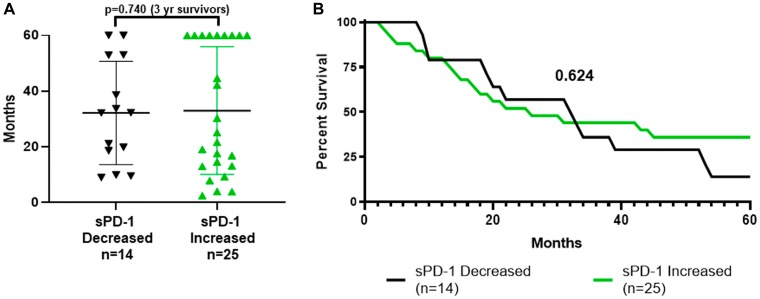
Survival by whether sPD-1 increased or decreased between week-0 and week-4. (**A**) shows distribution of survival with mean and standard deviation bars for the 14 patients whose sPD-1 decreased and for the 25 patients whose sPD-1 increased between week-0 and week-4. The proportions who survived more than three years, 5/14 vs 11/25, did not differ (*p =* 0.740, Fisher Exact Test). (**B**) shows the actual survival curves (all patients followed to death or five years with none lost to follow-up). There was no difference between the curves (*p =* 0.624, Mantel-Haenszel log rank-test). sPD-1 = soluble programmed cell death protein-1.

sPD-1 levels decreased in 11 patients and increased in 25 (Tables 1, 2, Figure 1). Changes in sPD-1 (increased versus decreased) were not predictive of survival as shown by dot plot distribution (Figure 4A) and survival curves (Figure 4B). In addition, changes in sPD-1 were not predictive of survival for either treatment arm (Tables 1, 2, Figure 1A). Changes in sPD-1 levels were also not predictive of survival for the 16 TCV-treated and 7 DCV-treated patients who survived less than three years (Tables 1, 2, Figure 1B), nor for 6 TCV-treated and the 10 DCV-treated patients who survived more than three years (Tables 1, 2, Figure 1C). For each of these cohorts the median and mean levels of sPD-1 were unchanged between week-0 and week-4 (Table 3). Thus, changes in sPD-1 levels were not predictive of survival in either treatment arm or any subset.

### Additional analyses by treatment arm suggested that a combination of baseline sPD-1 levels and changes in sPD-1 levels might be predictive for survival

**Figure 5:**
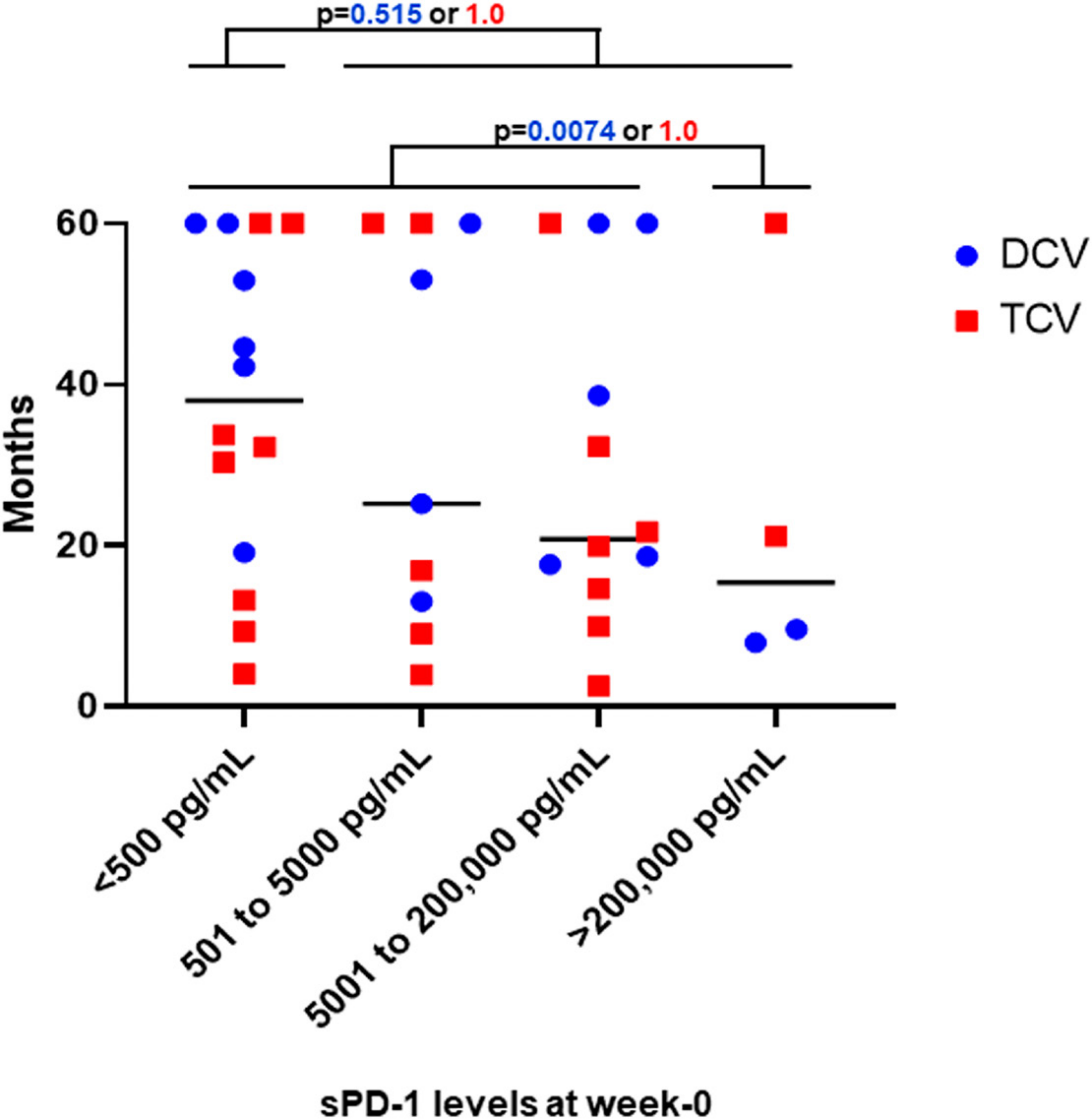
Survival distribution for all patients by treatment. The 39 patients are grouped by four different ranges of baseline sPD-1 levels. There was no difference in survival based on week-0 baseline sPD-1 ranges. Medians for all patients in each sPD-1 range are shown by black bar. Survivals for individual patients are shown for each of the 17 DCV-treated (blue) and each of the 22 TCV-treated patients (red). For DCV-treated patients who had baseline sPD-1 levels less than 200,000 pg/mL, 15/15 survived more than one year compared to 0/2 who had baseline sPD-1 levels greater than 200,000 pg/mL (*p =* 0.0074, Fisher Exact Test). For TCV-treated patients who had baseline sPD-1 levels less than 200,000 pg/mL, 14/20 survived more than one year compared to 2/2 who had sPD-1 levels greater than 200,000 at baseline (*p =* 1.00 Fisher Exact Test). For DCV-treated patients who had baseline sPD-1 levels less than 500 pg/mL, 6/6 survived more than one year compared to 9/11 DCV-treated patients who had baseline sPD-1 levels greater than 500 pg/mL (*p =* 0.515, Fisher Exact Test). For TCV-treated patients who had baseline sPD-1 levels less than 500 pg/mL, 6/8 survived more than one year compared to 10/14 who had sPD-1 levels greater than 200,000 pg/mL at baseline (*p =* 1.00 Fisher Exact Test). sPD-1= soluble programmed death protein-1.

Extremely high baseline sPD-1 levels were associated with poor survival in DCV-treated patients, but not TCV-treated. The association between baseline sPD-1 levels and survival of individual patients for all patients and by treatment are depicted in Figure 5. There were four patients with baseline sPD-1 levels greater than 200,000 pg/mL (Table 1, 2, Figure 5). Two of these patients were DCV-treated; both survived less than 10 months. Two were TCV-treated; one survived 21 months, and the other survived 5 years. The 5-year survivor was a 69-year-old man who entered the study as a recurrent stage 3 patient without measurable disease. He received all eight doses of TCV but progressed with a small bowel metastasis during treatment. This was resected, and during the following year he was treated with ipilimumab followed by resection of another small bowel recurrence. He received no subsequent treatment but remained disease-free the final three years of follow-up.

Figure 5 shows that all 15 DCV-treated patients with a baseline sPD-1 level of less than 200,000 pg/mL survived more than one year, in contrast to the two DCV-treated patients with a baseline level of greater than 200,000 pg/mL, neither of whom survived a year (15/15 vs 0/2, *p =* 0.0074). Among TCV-treated patients with a baseline sPD-1 less than 200,000 pg/mL, the proportion surviving one year was 14/20 compared to 2/2 for patients with a baseline sPD-1 greater than 200,000 pg/mL (*p =* 1.00) (Figure 5).

Relatively low levels of sPD-1 were associated with good survival in DCV-treated patients. Among DCV-treated patients, in the cohort with baseline sPD-1 levels less than 500 pg/mL, 5/6 survived more than 3 years compared to 5/11 with baseline sPD-1 levels greater than 500 pg/mL (*p =* 0.304). No such associations were evident for TCV-treated patients. For TCV-treated patients, in the cohort with baseline sPD-1 levels less than 500 pg/mL, 2/8 survived more than 3 years compared to 4/14 with baseline sPD-1 levels greater than 500 pg/mL (*p =* 1.00) (Figure 5). However, extremely low levels of sPD-1 were associated with good survival in both arms. There were 2/22 patients in the TCV-treated group with baseline sPD-1 levels less than 100 pg/mL; they survived 30 and 60+ months (Table 2). There were 3/17 in the DCV-treated group with such low levels, and their survivals were 42, 45, and 60+ months (Table 1). Thus, the five patients with the lowest baseline sPD-1 levels did relatively well regardless of which vaccine they received. None of these patients ever received anti-PD-1 or anti-PD-L1 therapy.

### A decrease in sPD1 levels was associated with longer survival in DCV-treated patients, but not TCV-treated

In terms of decreases in sPD-1 levels after three injections, there was no difference in the proportion of DCV-treated patients compared to TCV-treated patients (7/17 vs 7/22, *p =* 0.74) (Tables 1, 2, Figure 1A). However, among the patients who had a decline in sPD-1 between week-0 and week-4, 5/7 DCV-treated patients survived three years (Table 1), compared to 0/7 TCV-treated patients (*p =* 0.021) (Table 2). This observation combined with the associations of survival with very low and very high baseline sPD-1 levels led to the creation of two cohorts defined by a combination of baseline sPD-1 levels and changes in sPD-1 between week-0 and week-4.

### Classification using a combination of baseline sPD-1 and change in sPD-1 was predictive of survival for DCV-treated patients

**Figure 6:**
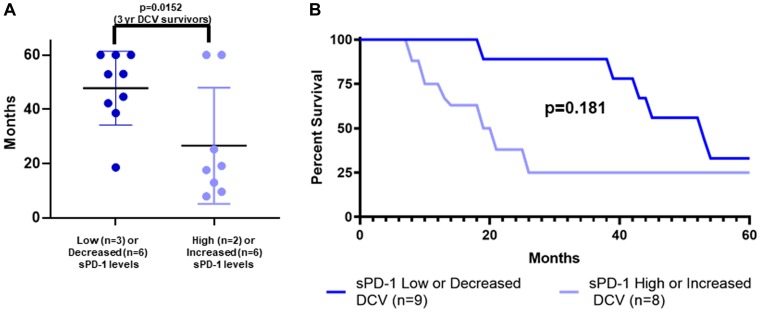
sPD-1 levels and survival of metastatic melanoma patients treated with autologous dendritic cell vaccine. Survival is shown by individual patients displaying mean with standard deviation (**A**) and actual survival curves (**B**) for two cohorts of DCV-treated patients. All patients were followed to death or for five years with none lost to follow-up. The two cohorts were defined by baseline levels of sPD-1 and changes in sPD-1 levels between week-0 and week-4. In DCV-treated patients, having sPD-1 level less than 100 pg/mL at baseline, or sPD-1 less than 200,000 pg/mL and a decrease in sPD-1 from week-0 to week-4 (sPD-1 low/decreased), was predictive of longer survival compared to sPD-1 level of greater than 200,000 pg/mL or greater than100 pg/mL and an increase in sPD-1 between week-0 and week-4 (sPD-1 high/increased). (A) dot plot: survival greater than 3 years in 8/9 vs 2/8 (*p =* 0.0152, Fisher Exact Test). (B) survival curves: median OS 48.8 vs 17.6 months (*p =* 0.182, Mantel-Haenszel log rank test). sPD-1=soluble programmed death protein-1, DCV=dendritic cell vaccine.

Patients were classified by a combination of baseline sPD-1 levels and change in sPD-1 levels between week-0 and week-4. Specifically, one cohort (sPD-1 low/decreased) was defined by a baseline sPD-1 of less than 100 pg/mL, or less than 200,000 pg/mL but with a decrease in sPD-1 between week-0 and week-4. The second cohort (sPD-1 high/increased) was defined by a baseline sPD-1 of greater than 200,000 pg/mL, or an increase in sPD-1 between week-0 and week-4. Figure 6 shows the survival of DCV-treated patients individually by dot plot (Figure 6A) and collectively by survival curves (Figure 6B), based on these classifications. Those in the sPD-1 low/decreased group had better survival. Figure 6A shows that 9/17 (52.9%, 95% CI 29.2% to 76.6%) of DCV-treated patients had a baseline sPD-1 level < 100 pg/mL, or a week-0 level between 100 pg/mL and 200,000 pg/mL and a subsequent decrease in sPD-1 levels; their median survival was more than four years with 8/9 surviving beyond three years. In contrast, Figure 6A also shows that the 8/17 DCV-treated patients in the sPD-1 high/increased group had a median survival of less than two years, and only 2/8 survived beyond three years (*p =* 0.0152). Thus, among the 17 DCV-treated patients, the classification using a combination of baseline sPD-1 and changes in sPD1 correctly classified 8/10 (80%) of 3-year survivors, and 6/7 (86%) of patients who survived less than three years.

The one false positive patient who survived less than 3-years was a 56-year-old female with measurable metastatic disease and elevated lactic dehydrogenase at the time of vaccine treatment. Her sPD-1 was 57,587 pg/mL at baseline and decreased to 19,160 at week-4 (patient #14, Table 1). She received all eight DCV doses, but was found to have progressive disease prior to receiving the last dose. She subsequently was treated with a combination of low-doses of GM-CSF, interleukin-2 and interferon alpha, then a combination of temozolomide, docetaxel, thalidomide, and sorafenib, and finally albumin-bound paclitaxel.

There were two false negative cases, that is, patients for whom the sPD-1 model predicted survival less than three years, but both were still alive five years later. The first was a 46-year-old male who was disease-free after resection of recurrent stage III disease. His sPD-1 was 42,393 pg/mL at baseline and increased to 52,570 pg/mL at week-4 (patient #13, Table 2). Two years after finishing the eight vaccine doses, he had a solitary lung metastasis that was treated with localized intensity modulated high-dose radiation. He remained disease-free thereafter. The other false negative was a 55-year-old male who had experienced local recurrence of ocular melanoma and liver metastases prior to receiving the vaccine, but had no measurable disease at the time of enrollment. His baseline sPD-1 was 7,645 pg/mL and increased to 10,340 pg/mL at week-4 (patient #11, Table 2). He received all eight doses but developed new liver metastases about 10 months after completing the vaccine. He subsequently responded well to high-dose interleukin-2 but progressed, then responded well to anti-PD-1 therapy, but again progressed, and was receiving ipilimumab for progressive disease at the time of 5-year follow-up. He was the only patient among the 39 to have received anti-PD-1 therapy during the course of the trial and follow-up.

**Figure 7:**
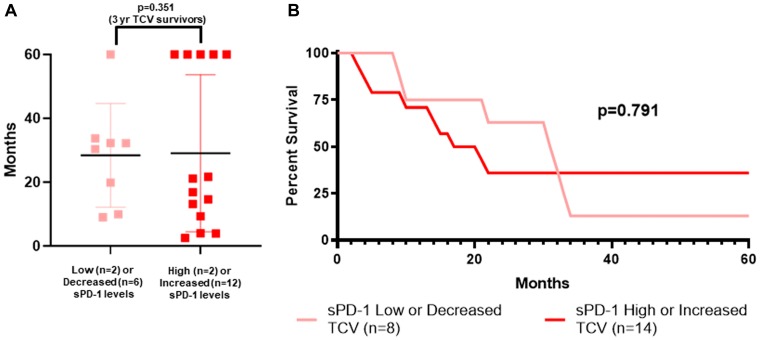
sPD-1 and survival of metastatic melanoma patients treated with autologous tumor cell vaccine. Survival is shown by individual patients displaying mean with standard deviation (**A**), and actual survival curves (**B**) for two cohorts of TCV-treated patients. All patients were followed to death or for five years with none lost to follow up. The two cohorts were defined by baseline levels of sPD-1 and changes in sPD-1 levels between week-0 and week-4. In TCV-treated patients, having sPD-1 level less than 100 pg/mL at baseline, or sPD-1 less than 200,000 pg/mL and a decrease in sPD-1 from week 0 to week 4 (sPD-1 low/decreased), was not predictive of longer survival compared to sPD-1 level of greater than 200,000 pg/mL or greater than 100 pg/mL and an increase in sPD-1 between week-0 and week-4 (sPD-1 high/increased). (A) dot plot: survival greater than 3 years in 5/14 vs 1/8 (*p =* 0.351 Fisher Exact Test). (B) survival curves: median OS 30.3 vs 16.9 mos (*p =* 0.791, Mantel-Haenszel log rank test). sPD-1=soluble programmed death protein-1, TCV=tumor cell vaccine.

At first glance it appeared that perhaps TCV-treated patients were more likely to survive three years if their sPD-1 level increased between week-0 and week-4 (5/14) as opposed to decreasing (1/8), but this difference was not significant (*p =* 0.351) (Table 3, Figures 1B, 1C). Figure 7 shows the survival of TCV-treated patients, individually by dot plot (Figure 7A) and collectively by survival curves (Figure 7B) based on the same criteria used to classify DCV-treated patients. There were 8/22 TCV-treated patients in the sPD-1 low/decreased cohort. Their probability of surviving more than three years was no different than for those TCV-treated who were classified as sPD-1 high/increased (Figure 7). In TCV- treated patients, the sPD-1 criteria correctly classified only 1/6 (17%) of 3-year survivors and 10/16 (62%) of patients who survived less than three years.

## DISCUSSION

The most interesting aspect of this analysis was the finding that in DCV-treated patients, the “sPD-1 low/decreased” criteria defined by a baseline sPD-1 level less than100 pg/mL or a baseline level less than 200,000 pg/mL with a subsequent decrease in sPD-1 after three weekly vaccinations, correctly classified 80% of 3-year survivors and 86% of patients who survived less than three years. Similar associations were not seen in TCV-treated patients. In addition to the predictive value of very low or very high baseline sPD-1 levels in DCV-treated patients, in the intermediate range between 100 pg/mL and 200,000 pg/mL, a decrease in sPD-1 levels following vaccination was associated with better survival among DCV-treated patients, but not TCV-treated patients. This is additional evidence that the immunological effects of these two patient-specific vaccines are quite different, and likely explain the differences in survival between the treatment arms [12].

The major strength of this study is that paired blood samples to measure sPD-1 were obtained from patients enrolled in a randomized trial with long-term survival data for correlations, with all patients followed until death or up to five years with no patients lost to follow-up. Weaknesses of the study are the relatively small number of patients sampled, and not having paired samples for 100% of patients enrolled in the trial.

This preliminary data suggests that baseline sPD-1 levels could be useful as a prognostic marker to exclude patients with little chance of benefitting from treatment with DCV, and that combining baseline sPD-1 levels with changes in sPD-1 levels after vaccination could be useful as a predictive marker of survival; and, therefore possibly could be a surrogate marker for survival in trials of metastatic melanoma patients treated with DCV. Having a surrogate marker for survival would be extremely useful for rapidly predicting benefit from DCV therapy in the context of a clinical trial. Excluding patients with very high baseline sPD-1 levels might be useful to enrich for a patient-population that has a better chance of benefitting from such a vaccine. However, because of the nature of this analysis and the small numbers of patients analyzed, the findings must be interpreted as preliminary and hypothesis-generating rather than definitive, and should be explored prospectively in many more patients. It is unclear whether this sPD-1 classification would be useful with any other vaccine or in other tumor types.

The findings in this study did not support baseline sPD-1 levels as a prognostic marker for this population of metastatic melanoma patients who enrolled in a clinical trial testing patient-specific autologous cell-based vaccines. For all 39 patients, there was no difference in mean or median levels at baseline or four weeks later after the first three vaccine injections (Figure 1, Table 3). However, the associations between sPD-1 levels and survival were quite different in DCV-treated patients compared to TCV-treated patients, even though samples were not analyzed for the two TCV-treated patients who had the worst outcome among all patients, nor a DCV-treated patients who was one of the 12 patients in the randomized clinical trial who survived five years [12]. Being classified as sPD-1 low/decreased appeared to have predictive significance for patients treated with DCV, but not TCV. The two DCV-treated patients with the highest levels of sPD-1 at baseline had relatively poor survival.

There have been a few other reports examining sPD-1 levels in various cancers with no consistent prognostic or predictive patterns [16–21]. In one study, higher levels of sPD-1 were associated with longer progression-free survival and OS in 38 patients with non-small cell lung cancer who were being treated with erlotinib, an inhibitor of mutated epidermal growth factor receptor [17]. Among patients with hepatitis B virus, those who had an elevated sPD-1, had a higher risk of developing hepatocellular cancer [18]. Among 120 patients with hepatocellular cancer, those who had higher sPD-1 levels had a worse prognosis [19]. In 41 patients with advanced pancreatic cancer, sPD-1 levels were not correlated with outcome [21]. In that study, levels of sPD-1 and sPD-L1 were closely correlated and elevated in association with an elevated c-reactive protein, suggestive of systemic inflammation, and tumor infiltration with T cells, which was consistent with an active anti-tumor immune response that was being blunted. In that trial, the median sPD-1 level was only 117 pg/mL with a range from 40 to 26,000 pg/mL using an ELISA with chemoluscent detection. While sPD-1 was detectable in the serum of all patients analyzed, sPD-L1 was below the lower limit of detection in 15/41.

Does the data from the vaccine trial make sense immunologically? It may in terms of concepts of “immune deserts” and checkpoint inhibition of activated immune responses [22, 23]. Patients with no immune response would be expected to have very low levels of sPD-1 and might benefit from an effective vaccine given alone, concurrently with, or preceding treatment with anti-PD-1 or anti-PD-L1. All three DCV-treated patients with a baseline sPD-1 less than 100 pg/mL survived at least 3.5 years, and none received treatment with anti-PD-1 or anti-PD-L1. Patients with a very high sPD-1 level might not benefit from induction of additional immune responses to more antigens, or increased responses to recognized antigens, but might benefit from anti-PD-1 or anti-PD-L1 therapy to unleash the previously suppressed immune responses. Those patients with levels in between the extremes likely would benefit from the combination of a vaccine with anti-checkpoint inhibitor therapy [23–25]. There was much better survival in DCV-treated patients, which we believe is because the DCV is more effective than the TCV as a therapeutic vaccine approach in cancer patients, because of the *ex vivo* loading of the cellular antigens onto dendritic cells [11, 12, 26, 27].

The data may also make sense immunologically because of the potential association between sPD-1 and suppression of immune responses. We believe the levels of sPD-1 and changes in sPD-1 are a reflection of Th1 immune responses and their suppression by the PD-1/PD-L1 axis. A recently induced or augmented immune response may be suppressed by increases in PD-1 expression by T lymphocytes that may result in increases in sPD-1. A decrease in sPD-1 or a lack of increase of sPD-1 following DCV may be indicative of lack of inhibition of a new effective immune response via the PD-1/PD-L1 regulatory axis.

## MATERIALS AND METHODS

### Patients and blood samples

Blood samples were obtained from metastatic melanoma patients who were enrolled in a randomized phase II trial that tested an autologous tumor cell vaccine (TCV) and an autologous dendritic cell vaccine (DCV) (NCT00948480) [11, 12]. The trial was conducted per the doctrine of Helsinki and all subjects gave written informed consent to participate. The details regarding the vaccine products and patient outcomes were previously published [11, 12]. Briefly, TCV consisted of irradiated self-renewing cancer cells, and DCV consisted of autologous dendritic cells loaded with antigens from self-renewing autologous cancer cells. Such cancer cells have characteristics of tumor initiating cells including cancer stem cells and progenitor cells [28, 29]. DC were derived from peripheral blood mononuclear cells obtained during a leukapheresis procedure. Both cellular vaccines were suspended in granulocyte-macrophage colony stimulating factor (GM-CSF) for subcutaneous injections that were planned for weeks 1, 2, 3, 8, 12, 16, 20, and 24.

The eligibility criteria, patient characteristics, early survival and long-term survival outcomes for the patients enrolled in the randomized trial have been published [11, 12]. Basically patients had to have stage 4 or recurrent stage 3 melanoma, had undergone resection of one or more lesions from which a short-term cell line had been established, had a Karnofsky performance status of 70 or greater at the time of randomization, and were referred by their managing physician for randomization to TCV or DCV. As previously reported, the original plan was to enroll 200 patients, but enrollment was discontinued early at a time when 42 patients had been randomized [11]. As recently published, long-term follow up of the randomized trial confirmed that DCV was associated with longer median survival (43.4 vs 20.5 months), better actual survival at three years (61% vs 25%, *p =* 0.018), and a 70% reduction in the risk of death (*p =* 0.0053) [12].

As a component of the trial, patients had blood samples collected one week before starting the vaccine injections, and one week after the third weekly injection. The focus of this report are the patients for whom blood samples were available from both week-0, one week prior to the first injection, and week-4, one week following the third injection. Paired samples were available for 39 of the 42 patients. The three patients for whom blood samples were missing were two TCV-treated patients who did not have week-4 blood samples collected because of rapidly progressive disease, and one DCV-treated patient who survived more than five years, but rescinded permission to test his blood. Even without the survival data from those three patients, the proportion surviving three years was still greater among DCV-treated patients than TCV-treated patients (10/17 versus 6/22, *p =* 0.047, Fisher Exact Test).

### Soluble PD-1 levels

Paired cryopreserved serum samples from week-0 and week-4 from each patient were analyzed using a quantitative, multiplex enzyme-linked immunosorbent assay per good laboratory practice (GLP) standards (Raybiotech, Inc., Norcross, GA). Results for sPD-1 were reported in picograms/milliliter (pg/mL).

### Statistics

Proportions were compared using the Fisher exact test. Because of the broad data distribution, grouped sPD-1 levels were compared by the non-parametric Mann–Whitney *U* test. Paired comparisons of week-0 versus week-4 sPD-1 levels were made by the Wilcoxon signed-rank test. Differences in survival curves were compared using the Mantel-Haenszel log-rank test. For all tests, the significance level was 0.05 and hypothesis tests were two-tailed.

## CONCLUSIONS

The combination of sPD-1 levels at baseline and change in sPD-1 levels following three weekly vaccines, appeared to predict long-term survival in patients with metastatic melanoma who were treated with autologous DCV. However, the retrospective analysis and small patient numbers mean that this is only hypothesis-generating and requires confirmation in larger trials.

## Abbreviations

DC: dendritic cell
DCV: dendritic cell vaccine
OS: overall survival
PD-1: programmed cell death protein-1
PD-L1: programmed cell death ligand-1
sPD-1: soluble programmed cell death protein-1
sPD-L1: soluble programmed cell death protein ligand-1
TCV: tumor cell vaccine
